# Fabrication and Characterization of Electrospun Cardiac Patches Functionalized with Microbiota-Derived Postbiotics and Decellularized Neonatal Porcine Myocardial Extracellular Matrix for Cardiac Repair

**DOI:** 10.3390/polym18141789

**Published:** 2026-07-22

**Authors:** Buket Celik, Ahmet Ceylan, Okan Ali Aksoy, Berk Alp Goksel, Mehmet Fazıl Tolga Soyal, Fadime Kiran

**Affiliations:** 1 Pharmabiotic Technologies Research Laboratory, Department of Biology, Faculty of Science, Ankara University, 06100 Ankara, Türkiye; bukketcellik@gmail.com; 2 Department of Biology, Graduate School of Natural and Applied Sciences, Ankara University, 06110 Ankara, Türkiye; 3 Department of Histology and Embryology, Faculty of Veterinary Medicine, Ankara University, 06070 Ankara, Türkiye; ceylan@ankara.edu.tr; 4 Animal Breeding and Research Center, Gülhane Health Institute, University of Health Sciences, 06010 Ankara, Türkiye; okanali.aksoy@sbu.edu.tr (O.A.A.); berkalp.goksel@sbu.edu.tr (B.A.G.); 5 Department of Cardiovascular Surgery, Losante Hospital, 06830 Ankara, Türkiye; tolga@soyal.org; 6 School of Medicine, Atilim University, 06830 Ankara, Türkiye

**Keywords:** cardiac tissue engineering, electrospinning, decellularized extracellular matrix, postbiotics, myocardial repair

## Abstract

Myocardial infarction remains a leading cause of heart failure owing to the limited regenerative capacity of adult cardiac tissue, underscoring the need for biomimetic therapeutic platforms that combine structural support with biological functionality. Accordingly, this study aimed to develop a multifunctional electrospun cardiac patch by integrating decellularized neonatal porcine myocardial extracellular matrix (dECM), gelatin, and microbiota-derived postbiotics for cardiac tissue engineering. The fabricated patches were comprehensively characterized in terms of their morphology, mechanical properties, biodegradation behavior, antibacterial activity, antioxidant capacity, and *in vitro* biocompatibility. Postbiotics derived from *Lactiplantibacillus plantarum* EIR/IF-1 exhibited potent antimicrobial activity against methicillin-resistant *Staphylococcus aureus*, strong antioxidant capacity, and significant anti-inflammatory activity through the suppression of pro-inflammatory mediators and upregulation of IL-10 expression. Moreover, they protected H9c2 cardiomyoblasts from oxidative stress, promoted *COL1A1* expression, and supported ECM remodeling. The fabricated electrospun cardiac patches exhibited a homogeneous nanofibrous architecture, mechanically suitable properties (Young’s modulus ~4 MPa), controlled biodegradation over 7 days, favorable cell viability, and maintained the biological functionality of the incorporated postbiotics. Overall, the synergistic integration of tissue-specific dECM and microbiota-derived postbiotics yielded a multifunctional biohybrid cardiac patch with favorable structural and biological properties, supporting its potential as a promising platform for myocardial regeneration and next-generation cardiac tissue engineering.

## 1. Introduction

Cardiovascular diseases (CVDs) remain the leading cause of global mortality, with annual deaths rising from 13.1 million in 1990 to 19.2 million in 2023, comprising approximately one-third of all global deaths. Projections indicate that, by 2050, CVD-related mortality is expected to rise to approximately 35.6 million deaths annually, accompanied by a global prevalence of around 1.14 billion cases, further underscoring the escalating burden of CVDs worldwide [1]. Among CVDs, myocardial infarction (MI) constitutes one of the leading causes of morbidity and mortality worldwide and is a major contributor to the development of chronic heart failure (HF). The irreversible loss of cardiomyocytes following ischemic injury initiates a complex cascade of inflammatory responses, oxidative stress, extracellular matrix (ECM) remodeling, and fibrotic scar formation, ultimately resulting in progressive deterioration of cardiac function [2]. Although current clinical interventions, including pharmacological therapy, percutaneous coronary intervention, coronary artery bypass grafting, and heart transplantation, have substantially improved patient survival, these therapeutic approaches primarily alleviate symptoms or restore coronary perfusion without effectively regenerating the damaged myocardium. Consequently, the limited intrinsic regenerative capacity of adult cardiac tissue remains a major obstacle to complete functional recovery following MI, underscoring the need for innovative regenerative strategies capable of restoring both the structural and functional integrity of the injured myocardium [3].

In recent years, cardiac tissue engineering has emerged as a promising therapeutic approach for myocardial regeneration [2]. Among the various strategies investigated, cardiac patches have attracted considerable attention because they provide temporary mechanical support to the infarcted myocardium while simultaneously serving as platforms for cell retention, therapeutic molecule delivery, and tissue remodeling [2,3,4,5]. Accordingly, recent advances in cardiac tissue engineering have shifted the design paradigm from passive structural scaffolds toward multifunctional bioactive platforms that combine mechanical reinforcement with biological activity to simultaneously modulate inflammation, oxidative stress, angiogenesis, and tissue regeneration [4]. In this context, electrospinning has emerged as one of the most versatile fabrication techniques because it enables the production of highly porous nanofibrous scaffolds that closely resemble the architecture of the native ECM while providing a large surface area for cell attachment and efficient incorporation of bioactive compounds [6]. Consequently, electrospun cardiac patches incorporating growth factors, stem cells, extracellular vesicles, conductive nanomaterials, or naturally derived biomolecules have demonstrated encouraging outcomes in improving cardiomyocyte survival, vascularization, electrical coupling, and myocardial regeneration [3,7,8,9]. Among naturally derived biomaterials, decellularized ECM (dECM) has emerged as one of the most promising biomaterials for cardiac tissue engineering because it preserves the native biochemical composition, ultrastructure, and tissue-specific biological cues of the myocardium [10]. Numerous studies have demonstrated that dECM-based scaffolds enhance cell adhesion, proliferation, differentiation, and constructive tissue remodeling while exhibiting excellent biocompatibility and reduced immunogenicity [11,12]. Nevertheless, most reported dECM-based cardiac patches primarily provide structural support [13] and have limited capability to actively regulate the inflammatory and oxidative microenvironment following myocardial infarction [4,5].

In parallel with advances in biomaterial design, increasing evidence has highlighted the critical role of the gut–heart axis in the pathogenesis and progression of cardiovascular diseases [14,15]. Alterations in gut microbiota composition have been associated with chronic inflammation, oxidative stress, immune dysregulation, and adverse cardiac remodeling through the production of various microbial metabolites [16]. Consequently, microbiota-derived bioactive molecules have recently emerged as attractive therapeutic candidates capable of modulating multiple pathological pathways involved in myocardial injury. Among these metabolites, postbiotics have gained considerable attention because they consist of non-viable microbial cells, cell components, and biologically active metabolites that retain many of the beneficial effects of probiotics while offering superior stability, safety, and manufacturing consistency [17,18]. Accumulating evidence demonstrates that postbiotics exhibit diverse biological activities, including antimicrobial, antioxidant, anti-inflammatory, immunomodulatory, and cytoprotective effects, making them promising candidates for regenerative medicine applications [19,20,21,22]. These multifunctional properties are particularly relevant for myocardial repair, where excessive inflammation and oxidative stress constitute major determinants of cardiomyocyte death, impaired ECM remodeling, and progressive ventricular dysfunction. Nevertheless, despite the rapidly growing interest in postbiotics across various biomedical fields [22], their application in cardiac tissue engineering remains remarkably limited. To date, studies incorporating postbiotics into electrospun biomaterials have been largely restricted to wound healing and antimicrobial dressings [23,24,25,26,27], whereas their integration into bioactive cardiac patch platforms has rarely been explored. Therefore, there remains a significant knowledge gap regarding the development of multifunctional cardiac patches capable of simultaneously providing tissue-specific structural support and microbiota-derived biological functionality. Addressing this gap may offer a novel strategy for improving the therapeutic performance of cardiac patches by targeting multiple pathological mechanisms involved in myocardial infarction rather than focusing solely on structural tissue replacement.

In light of these considerations, integrating tissue-specific ECM with biologically active therapeutic agents represents a promising strategy for developing next-generation multifunctional cardiac patches capable of enhancing myocardial regeneration. While dECM provides a biomimetic structural microenvironment that supports cell adhesion, tissue remodeling, and cardiac repair, the incorporation of microbiota-derived postbiotics offers the potential to actively modulate inflammation, oxidative stress, and bacterial contamination, thereby addressing several key pathological processes associated with myocardial infarction. Therefore, the present study aimed to develop and comprehensively characterize a multifunctional electrospun cardiac patch composed of gelatin, decellularized neonatal porcine myocardial ECM, and microbiota-derived postbiotics derived from *Lactiplantibacillus plantarum* EIR/IF-1 (NCBI GenBank accession no. OP810909.1). The strain was originally isolated from the microbiota of breast-fed infant feces and selected based on its well-established probiotic profile and multifunctional bioactive properties [21,28]. The corresponding postbiotic preparation was comprehensively characterized using HPLC, GC-MS, and LC-MS/MS analyses, revealing a diverse metabolite profile composed predominantly of organic acids (particularly lactic acid), fatty acid methyl esters (FAMEs), and vitamins, which are considered to contribute to its antimicrobial, antioxidant, and immunomodulatory activities [29]. Neonatal myocardial tissue was intentionally chosen as the ECM source because it provides a developmentally enriched regenerative microenvironment containing structural proteins, matricellular components, and bioactive signaling molecules that have been shown to better support cardiomyocyte survival, tissue remodeling, and regenerative processes than adult cardiac ECM [30,31].

To the best of our knowledge, this is the first study to integrate microbiota-derived postbiotics with decellularized neonatal porcine myocardial ECM into an electrospun cardiac patch specifically designed for myocardial tissue engineering. This unique combination integrates tissue-specific structural biomimicry with microbiota-derived biological functionality within a single electrospun platform, enabling the simultaneous provision of mechanical support together with antibacterial, antioxidant, anti-inflammatory, and ECM-regulating activities. Collectively, this biohybrid strategy expands the current design paradigm of electrospun cardiac patches beyond passive structural replacement and represents a promising platform for next-generation myocardial regeneration and regenerative cardiovascular medicine.

## 2. Materials and Methods

### 2.1. Decellularization of Neonatal Porcine Myocardial Tissue

Porcine hearts were harvested from 1-week-old Yorkshire domestic pigs (*Sus scrofa domesticus*) (*n* = 4), obtained from a slaughterhouse affiliated with the Gülhane Experimental Animals Production and Research Center (Gülhane Health Institute, R&D Center Presidency). All procedures involving animal-derived tissues were conducted in accordance with institutional ethical standards and approved by the Local Ethics Committee for Animal Experiments of the University of Health Sciences (Approval No: ETH-2023/05).

Neonatal porcine hearts were retrieved within 2 h following animal sacrifice, and rapidly transferred to the laboratory under sterile and cold-chain conditions, then rinsed in 1× PBS (Sigma-Aldrich, St. Louis, MO, USA) for 1 h under magnetic stirring to remove blood components. Subsequently, atrial and ventricular regions were dissected to eliminate adipose and large vascular tissues, and the remaining myocardial tissue was mechanically minced into fragments smaller than 1 mm^3^ using sterile forceps. The obtained tissue fragments were then subjected to decellularization by incubation in 0.05% (*w*/*v*) sodium dodecyl sulfate (SDS; Sigma-Aldrich, St. Louis, MO, USA) solution for 24 h, ensuring complete immersion and continuous agitation. Following this step, samples were washed with distilled water and further treated with 0.1% (*w*/*v*) SDS solution under the same conditions. This decellularization cycle was continued for 24–72 h, depending on tissue size, until a visible whitening of the tissue was observed, with the solution replaced every 24 h to maintain efficacy. Once decellularization was achieved, the samples were rinsed again with distilled water and subsequently incubated in 1% (*v*/*v*) Triton X-100 (Sigma-Aldrich, St. Louis, MO, USA) solution for 1 h to facilitate removal of residual cellular debris. To ensure complete elimination of Triton X-100, samples were immersed in distilled water for 24 h, during which the water was frequently refreshed to enhance washing efficiency. Thereafter, residual chemical agents were removed by washing the samples three times with 0.9% (*w*/*v*) NaCl isotonic solution (Merck, Darmstadt, Germany). For further decontamination, tissues were incubated in a solution containing 4% ethanol (Merck, Darmstadt, Germany) and 1% peracetic acid (Sigma-Aldrich, St. Louis, MO, USA) for 5 h. Finally, the samples were thoroughly rinsed three times with distilled water, followed by gradual freezing and subsequent lyophilization using a freeze dryer (Büchi Labortechnik AG, Flawil, Switzerland). All procedures were performed under sterile conditions at 4 °C with continuous agitation.

### 2.2. Characterization of dECM

#### 2.2.1. Quantification of DNA Content

To evaluate the efficiency of decellularization in removing cellular components, the DNA content of native tissue and dECM samples was measured. Genomic DNA was extracted using the FavorPrep™ Tissue Genomic DNA Extraction Kit (Favorgen Biotech Corp., Ping-Tung, Taiwan) according to the manufacturer’s instructions. DNA concentration and purity were determined by measuring absorbance at 260/280 nm using an Epoch Microplate Reader (BioTek Instruments, Winooski, VT, USA).

#### 2.2.2. Histological Evaluation and Immunofluorescence Analysis

Histological evaluation of dECM and native tissue was performed on paraffin- embedded sections (5 μm). Tissue morphology and ECM organization were assessed using Hematoxylin and Eosin (H&E, Sigma-Aldrich, St. Louis, MO, USA) staining and Masson’s Trichrome (Sigma-Aldrich, St. Louis, MO, USA) staining. Stained sections were visualized using a light microscope (Olympus, Tokyo, Japan). For immunofluorescence analysis, samples were fixed in 10% neutral buffered formalin (Sigma-Aldrich, St. Louis, MO, USA) for 18 h, embedded in paraffin, and sectioned at 4 μm thickness. Non-specific binding was blocked using 10% normal goat serum (Thermo Fisher Scientific, Waltham, MA, USA) for 10 min at room temperature. Sections were then incubated with anti-COL1A1 primary antibody (Abcam, Cambridge, UK) overnight at 4 °C, followed by incubation with FITC-conjugated secondary antibody (Abcam, Cambridge, UK) for 30 min at 37 °C. Nuclei were counterstained with DAPI (Sigma-Aldrich, St. Louis, MO, USA), and imaging was performed using a fluorescence microscope (Olympus, Tokyo, Japan) [28].

#### 2.2.3. Fourier Transform Infrared (FTIR) Spectroscopy

The chemical characteristics of native myocardial tissue and decellularized dECM were evaluated by attenuated total reflectance Fourier transform infrared (ATR-FTIR) spectroscopy. FTIR spectra were recorded using an IR Affinity-1 spectrometer (Shimadzu, Kyoto, Japan) over the spectral range of 500–4000 cm^−1^ with a spectral resolution of 4 cm^−1^. Prior to sample analysis, background spectra were obtained using 100 scans to minimize atmospheric interference and improve spectral accuracy. The resulting spectra were used to compare the characteristic functional groups and assess the preservation of the biochemical composition following decellularization.

### 2.3. Extraction of Microbiota-Derived Postbiotics

*Lactiplantibacillus plantarum* EIR/IF-1, obtained from the Pharmabiotic Technologies Research Laboratory culture collection, was used for postbiotic production. A glycerol stock stored at −80 °C was activated by inoculation into De Man, Rogosa, and Sharpe (MRS; Merck, Darmstadt, Germany) broth (1% *v*/*v*) and incubated at 37 °C for 24 h. For production, the activated culture was inoculated (1% *v*/*v*) into 200 mL MRS broth and incubated at 37 °C for 24 h. The culture was then centrifuged at 15,000× *g* for 20 min, and the resulting supernatant was filtered through a 0.22 μm membrane filter (Sartorius, Göttingen, Germany) to obtain a cell-free fraction. The filtrate was frozen sequentially at −20 °C and −80 °C, followed by lyophilization under 0.120 mbar and −58 °C condenser temperature [21]. The obtained postbiotic powder was stored at −20 °C until further use.

### 2.4. Antibacterial Activity of Postbiotics

The antibacterial activity of postbiotics against Methicillin-resistant *Staphylococcus aureus* (MRSA, ATCC 43300) was evaluated using the agar well diffusion method, broth microdilution, and Live/Dead fluorescence staining assays. For the agar well diffusion assay [32], MRSA cell suspensions adjusted to 0.5 McFarland standard (bioMérieux, Marcy-l’Étoile, France) were spread onto Brain Heart Infusion (BHI; Merck, Darmstadt, Germany) agar plates, and 100 μL of postbiotic solution (100 mg/mL) was added into agar wells. Vancomycin discs (30 µg/disc; Oxoid, Hampshire, UK) were used as positive controls. To investigate whether the antibacterial activity was associated with antimicrobial peptides or acid-dependent metabolites, postbiotic samples were additionally treated with proteinase K (1 mg/mL; Sigma-Aldrich, St. Louis, MO, USA) or neutralized using sterile-filtered 1 N NaOH (Sigma-Aldrich, St. Louis, MO, USA) prior to application [33]. Following incubation at 37 °C for 24 h, inhibition zone diameters were measured. Minimum inhibitory concentration (MIC) values were also determined using the broth microdilution method in 96-well microplates (LP Italiana, Milan, Italy) according to the Clinical and Laboratory Standards Institute guidelines [34]. Lyophilized postbiotics were serially diluted (1–100 mg/mL) and incubated with MRSA cell suspensions adjusted to a 0.5 McFarland standard. After incubation at 37 °C for 24 h, bacterial growth was quantified spectrophotometrically at 600 nm using an Epoch Microplate Reader, and the lowest concentration showing no visible bacterial growth was defined as the MIC value. Bacterial viability after postbiotic treatment was further assessed using SYTO 9/propidium iodide (PI)-based Live/Dead fluorescence staining according to the manufacturer’s instructions (Thermo Fisher Scientific, Waltham, MA, USA). Fluorescence images were obtained using a ZEISS Primovert fluorescence microscope (Carl Zeiss AG, Oberkochen, Germany) at different incubation time points. In all experiments, MRS broth was used as the negative control, whereas untreated MRSA cell suspensions served as the positive control.

### 2.5. Antioxidant Potential of Postbiotics

The antioxidant capacity of postbiotics was evaluated by determination of total phenolic content, total flavonoid content, and free radical scavenging activity. Total phenolic content was quantified using the Folin–Ciocalteu colorimetric method with gallic acid (Sigma-Aldrich, St. Louis, MO, USA) as the reference standard [35], whereas total flavonoid content was determined by the aluminum chloride assay using quercetin (Sigma-Aldrich, St. Louis, MO, USA) as the standard compound [36]. In addition, free radical scavenging activity was assessed using the 2,2-diphenyl-1-picrylhydrazyl (DPPH, Sigma-Aldrich, St. Louis, MO, USA) according to the method described by Blois [37]. Briefly, postbiotic solutions were incubated with DPPH solution (120 μM) under dark conditions, and absorbance values were measured spectrophotometrically at 517 nm. Antioxidant activity was expressed as percentage inhibition, calculated using the formula [(Acontrol − Asample)/Acontrol] × 100, where A represents absorbance. In all assays, MRS medium was included as the blank control.

### 2.6. In Vitro Evaluation of the Cardioprotective Potential of Postbiotics

H9c2 rat cardiomyoblast cells (ATCC, CRL-1446) were employed as an in vitro model to assess the effects of postbiotics under inflammatory and oxidative stress conditions. H9c2 cells were cultured in Dulbecco’s Modified Eagle Medium (DMEM; Invitrogen, Carlsbad, CA, USA) supplemented with 10% fetal bovine serum (FBS; Sigma-Aldrich, USA) and 1% penicillin–streptomycin solution (Thermo Fisher Scientific, Waltham, MA, USA), and maintained at 37 °C in a humidified atmosphere containing 5% CO_2_. The culture medium was replaced every 2–3 days, and cells were passaged upon reaching 80–90% confluence. Prior to each experiment, cell density and viability were determined using the trypan blue exclusion (Sigma-Aldrich, St. Louis, MO, USA) by an automated cell counter (TC20, Bio-Rad Laboratories, Hercules, CA, USA). All experiments were conducted using cells between passages 5 and 10, and all cultures were routinely screened and confirmed to be free of mycoplasma contamination.

#### 2.6.1. Cytotoxicity Analysis

To determine the appropriate experimental concentrations of postbiotics, dose–response analyses were initially performed. Briefly, H9c2 cells were seeded at 1 × 10^4^ cells/well in 96-well plates and allowed to attach overnight. Cells were then treated with increasing concentrations of postbiotics (500–2500 µg/mL) for 24 h, while untreated cells served as controls. Cell viability was assessed using the MTT assay according to the method described by Mosmann [38]. Following treatment, MTT solution (5 mg/mL; Sigma-Aldrich, St. Louis, MO, USA) was added to each well and incubated for an additional 4 h at 37 °C. The resulting formazan crystals were dissolved in dimethyl sulfoxide (DMSO; Sigma-Aldrich, St. Louis, MO, USA), and absorbance was measured at 570 nm using an Epoch Microplate Reader. Based on the obtained results, the highest non-cytotoxic concentration of postbiotics was selected for subsequent experiments. Similarly, concentration-dependent cytotoxicity analyses were also performed for hydrogen peroxide (H_2_O_2_; Sigma-Aldrich, St. Louis, MO, USA) and LPS from *Escherichia coli* O111:B4 (LPS; Sigma-Aldrich, St. Louis, MO, USA) to establish oxidative stress and inflammatory models, respectively. H9c2 cells were exposed to different concentrations of H_2_O_2_ (100–1000 µM) and LPS (1–50 µg/mL), and cell viability was evaluated using the same MTT protocol.

#### 2.6.2. Effects of Postbiotics Against LPS-Induced Inflammation

To evaluate the anti-inflammatory potential of postbiotics, an in vitro inflammatory model was established in H9c2 cardiomyoblast cells using LPS. Briefly, H9c2 cells were seeded at a density of 1 × 10^6^ cells per well and pretreated with the highest non-cytotoxic concentration of postbiotics for 18 h, followed by stimulation with LPS for 6 h [39]. Untreated cells served as negative controls, whereas cells exposed to LPS alone were used as the inflammatory group. To investigate inflammatory signaling at the transcriptional level, total RNA was isolated from treated cells using PureZOL reagent (Bio-Rad Laboratories, Hercules, CA, USA), followed by cDNA synthesis (Bio-Rad Laboratories, Hercules, CA, USA) via reverse transcription. Quantitative real-time PCR (qRT-PCR) was performed using SYBR Green Master Mix (Bio-Rad Laboratories, Hercules, CA, USA), and the expression levels of *IL-1β*, *IL-6*, *TNF-α*, *NF-κB*, and *IKK-α* were normalized to *GAPDH* and calculated using the comparative Ct (2^−ΔΔCt^) method. Primers used in this study are listed in Table 1.

In addition, anti-inflammatory effects were further validated at the protein level by measuring TNF-α, IL-6, IL-10, and IL-1β concentrations in cell culture supernatants using commercially available ELISA kits (Mabtech AB, Nacka Strand, Sweden). Absorbance was recorded at 405 nm using an Epoch Microplate Reader, and cytokine concentrations were calculated based on standard curves.

#### 2.6.3. Effects of Postbiotics Against H_2_O_2_-Induced Oxidative Stress

An in vitro oxidative stress model was established in H9c2 cardiomyoblast cells. Briefly, cells were pretreated with postbiotics for 23 h and subsequently exposed to hydrogen peroxide (H_2_O_2_; IC_50_) for 1 h to induce oxidative stress conditions [40]. Untreated cells served as negative controls, whereas cells exposed to H_2_O_2_ alone represented the oxidative stress group. Viability of cells and apoptotic cell death were quantified by Annexin V–propidium iodide (PI) dual staining kit (BD Biosciences, San Jose, CA, USA). Following treatment, H9c2 cells were harvested using 0.25% trypsin (Thermo Fisher Scientific, Waltham, MA, USA), washed twice with cold PBS, and resuspended in 500 μL binding buffer. Cells were then incubated with 10 μL Annexin V for 60 min at 4 °C in the dark, followed by 5 μL PI staining for 5 min at room temperature. Apoptotic populations were analyzed using a flow cytometer (NovoCyte, ACEA Biosciences, San Diego, CA, USA), and data were processed using FlowJo software (version 10.5.3, Tree Star, San Carlos, CA, USA).

For biochemical analysis, cells were collected after the treatments at a density of 1 × 10^6^ cells/mL, washed twice with PBS, and centrifuged at 1500 rpm for 10 min. The resulting pellets were lysed by ultrasonic disruption to obtain cell lysates. Total superoxide dismutase (T-SOD), catalase (CAT), and malondialdehyde (MDA) levels were measured using commercial assay kits according to the manufacturers’ instructions (Elabscience Biotechnology Inc., Houston, TX, USA). Protein concentrations were determined using the Bradford/BCA assay (Pierce, Rockford, IL, USA).

#### 2.6.4. Effects of Postbiotics on Collagen Synthesis

The effects of postbiotics on collagen synthesis were evaluated at both gene expression and cellular levels. For gene expression analysis, total RNA was isolated from treated and control cells using a commercial RNA extraction kit, followed by reverse transcription to synthesize complementary DNA (cDNA). qRT-PCR was performed to determine the relative expression levels of *COL1A1* using specific primers, listed in Table 1. Gene expression data were analyzed as described in Section 2.6.2.

In parallel, the effects of postbiotics on collagen production and ECM remodeling were assessed using immunofluorescence staining. Briefly, H9c2 cells (1 × 10^6^ cells/mL) were seeded onto glass coverslips and incubated with or without postbiotics for 24 h. Following treatment, cells were fixed with 4% paraformaldehyde (Sigma-Aldrich, St. Louis, MO, USA) in phosphate-buffered saline (PBS) for 20 min. After fixation, samples were incubated with a primary antibody against COL1A1 (Abcam, Cambridge, UK), followed by incubation with a FITC-conjugated secondary antibody (Abcam, Cambridge, UK). Nuclei were counterstained with DAPI (Sigma-Aldrich, St. Louis, MO, USA). Slides were mounted using ProLong Gold Antifade Reagent (Thermo Fisher Scientific, Waltham, MA, USA) and visualized under a fluorescence microscope equipped with a Color View III cooled CCD camera. Fluorescence signals were captured using an excitation wavelength of 488 nm and an emission wavelength of 520 nm.

### 2.7. Fabrication of Electrospun Hybrid Cardiac Patches

dECM was enzymatically digested in 1 mg/mL porcine pepsin solution (Sigma-Aldrich, St. Louis, MO, USA) prepared in 0.01 N HCl (Merck, Darmstadt, Germany) under constant magnetic stirring at room temperature for 72 h. During this process, pepsin selectively cleaves the telopeptide regions of collagen, solubilizing the ECM into a pre-gel solution composed primarily of monomeric collagen and other ECM proteins. This enzymatic digestion step facilitates the formation of a homogeneous mixture by disrupting fibrillar aggregates. The digest was then neutralized by titration with 1 M NaOH at a volumetric ratio of 1:10, followed by adjustment of ionic strength and pH through the addition of 10× PBS at a 1:9 ratio. Upon neutralization, the resulting solution (with 1% and 2% dECM) was combined with gelatin (5%) and postbiotics (MIC-10) dissolved in hexafluoroisopropanol (HFIP; Haihang Industry Co., Ltd., Jinan, China). Electrospinning was performed using the NE300 electrospinning system (Inovenso, Istanbul, Türkiye) with optimized parameters of 20 kV voltage, 200 mm needle-to-collector distance, and 2 mL/h flow rate. Fabricated patches were crosslinked using glutaraldehyde (Merck, Darmstadt, Germany) vapor for 4 h.

### 2.8. Characterization of Electrospun Hybrid Cardiac Patches

#### 2.8.1. Morphological Characterization

The surface morphology and fiber architecture of the electrospun hybrid cardiac patches were evaluated using scanning electron microscopy (SEM; Leitz, Wetzlar, Germany). Prior to imaging, scaffold samples were fixed onto aluminum stubs using conductive carbon tape and sputter-coated with a thin layer of gold to improve conductivity. SEM analyses were performed under appropriate accelerating voltage conditions, and fiber organization, surface homogeneity, pore distribution, and structural integrity were qualitatively assessed. Fiber diameter and pore size were also quantified using ImageJ software (v1.54d; National Institutes of Health, Bethesda, MD, USA).

#### 2.8.2. Mechanical Characterization

The mechanical properties of the electrospun cardiac patches were systematically assessed using a universal tensile testing system (Shimadzu Corporation, Kyoto, Japan) for determining key mechanical parameters, including ultimate tensile strength, elongation at break, and Young’s modulus. Briefly, each scaffold specimen was securely fixed between the instrument grips and subjected to uniaxial tensile loading at a constant displacement rate of 1 mm/min until failure, with a maximum applied force limit of 50 N.

#### 2.8.3. Swelling Analysis

The swelling behavior of the electrospun cardiac patches was quantitatively evaluated to assess their water uptake capacity, which is critical for maintaining a hydrated wound environment. Briefly, scaffold samples were weighed to determine the initial mass (M_0_) and then immersed in 1× PBS at 37 °C. At predetermined time points (3, 12, 24, 48, and 72 h), samples were retrieved, and excess surface fluid was gently removed using filter paper to minimize measurement errors. The swollen mass (M_t_) was immediately recorded. The swelling ratio (S) was then calculated as the percentage increase relative to the initial weight using the following equation, where Wd represents the dry weight and Ws represents the swollen weight of the scaffold [41]:

$$S\left(\%\right)=\left[\frac{\left(M_{t}-M_{0}\right)}{M_{0}}\right]\times 100$$

#### 2.8.4. Biodegradation Analysis

For the biodegradation assay, scaffold samples were incubated in 3.2 U/mL collagenase type I solution (Sigma-Aldrich, St. Louis, MO, USA) at 37 °C to mimic enzymatic degradation conditions relevant to in vivo tissue remodeling. The initial dry weight (M_0_) was recorded prior to incubation. At predetermined time points (days 1, 3, 5, and 7), samples were collected, gently rinsed with deionized water, and excess surface moisture was removed before weighing to determine the residual mass (M_t_). The percentage of degradation (D) was then calculated using the following equation [41]:

$$D\left(\%\right)=\left[\frac{\left(M_{t}-M_{0}\right)}{M_{0}}\right]\times \;100$$

#### 2.8.5. Assessment of Biologic Activities

The antibacterial and antioxidant properties of the electrospun hybrid cardiac patches were evaluated against MRSA using the disk diffusion assay and by determining DPPH radical scavenging activity, respectively, as described above.

#### 2.8.6. In Vitro Biocompatibility Analysis

H9c2 cells were seeded onto the electrospun hybrid cardiac patches at a density of 1 × 10^5^ viable cells per construct and cultured for 5 days under standard incubation conditions (37 °C, 5% CO_2_, 90% humidity). After the incubation period, cell viability, adhesion, and morphological organization on scaffold surfaces were assessed. Cell viability on days 1 and 5 was evaluated using a Live/Dead fluorescence assay (Thermo Fisher Scientific, Waltham, MA, USA), and imaging was performed using a fluorescence microscope equipped with a digital imaging system [27].

### 2.9. Statistical Analysis

All experiments were performed in three independent biological replicates, each conducted in triplicate. Statistical analyses were conducted using GraphPad Prism software (version 8.0.2, GraphPad Software, San Diego, CA, USA). Differences between the two groups were assessed using Student’s *t*-test, while multiple group comparisons were conducted using one-way analysis of variance (ANOVA). Data are expressed as mean ± standard deviation (SD), and a value of *p* < 0.05 was considered statistically significant.

## 3. Results

### 3.1. Decellularization and Characterization of Neonatal Porcine Myocardial dECM

**Figure 1:**
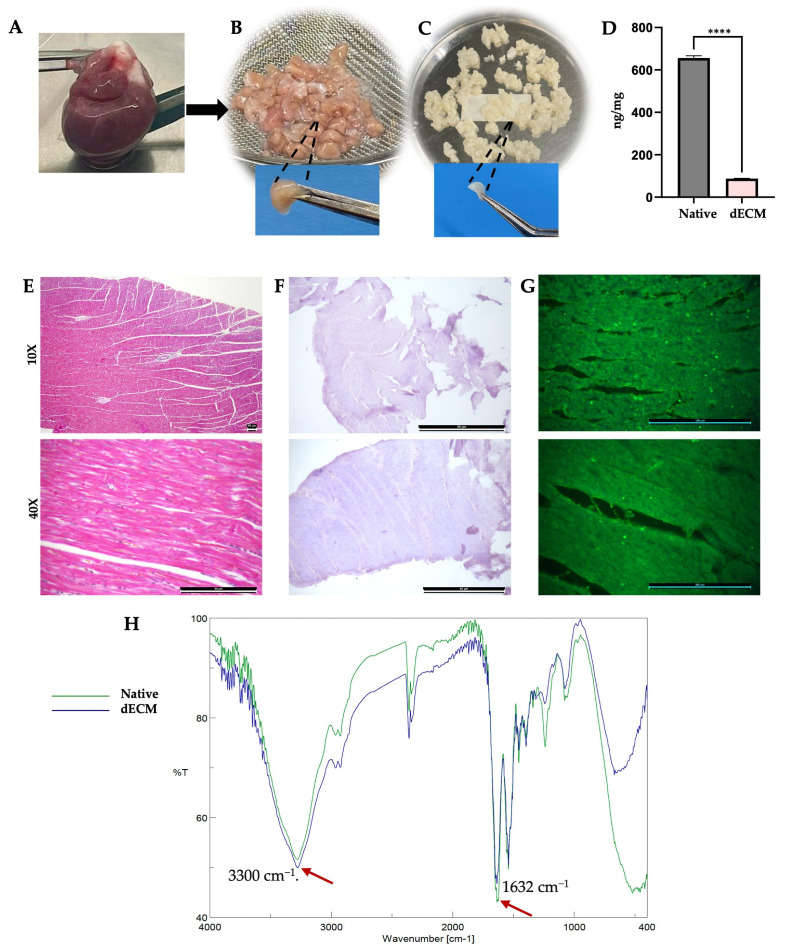
Decellularization and characterization of neonatal porcine myocardial ECM. (**A**) Surgically isolated neonatal porcine hearts. (**B**) Native myocardial tissue before decellularization. (**C**) Macroscopic appearance of dECM after decellularization. (**D**) DNA quantification of native myocardial tissue and dECM (**** *p* < 0.0001 versus native tissue). (**E**) Masson’s Trichrome staining of native myocardial tissue (scale bar = 50 μm). (**F**) H&E staining of dECM (scale bar = 50 μm). (**G**) Immunofluorescence staining of dECM showing COL1A1 (green) and DAPI (blue), scale bar = 100 μm). (**H**) Fourier Transform Infrared spectra of native myocardial tissue and dECM.

Neonatal porcine myocardial tissues obtained from surgically isolated hearts (Figure 1A) were subjected to decellularization protocols. Prior to decellularization, native myocardial tissue exhibited a characteristic reddish appearance due to the presence of cellular and blood-derived components within the tissue structure (Figure 1B). Following the decellularization, a distinct macroscopic color transition from red to whitish translucent was observed, indicating substantial removal of cellular constituents while preserving the ECM framework (Figure 1C). To further assess decellularization efficiency, the DNA contents of native myocardial tissue and dECM were quantified. Native myocardial tissue exhibited a DNA concentration of 656 ± 7.0 ng/mg, whereas the DNA content of dECM was significantly reduced to 46 ± 0.5 ng/mg following decellularization (*p* < 0.0001) (Figure 1D).

Histological analyses also demonstrated the preservation of ECM integrity after decellularization. Masson’s Trichrome staining of native myocardial tissue revealed well-organized collagen fibers, muscle fibers, and intact cellular structures (Figure 1E). In contrast, H&E staining of dECM samples demonstrated the absence of visible nuclei while maintaining the structural organization of the ECM, indicating efficient cellular removal without substantial disruption of tissue architecture (Figure 1F). Immunofluorescence analysis using anti-COL1A1 antibody further confirmed preservation of collagen content within the dECM (Figure 1G). Strong green fluorescence corresponding to collagen type I was observed throughout the dECM structure, whereas minimal DAPI-positive nuclei were detected, further supporting the successful elimination of cellular constituents while preserving the collagen-rich myocardial dECM structure.

To further evaluate the biochemical preservation of the ECM following decellularization, FTIR analysis was performed to compare the chemical characteristics of native myocardial tissue and dECM. As shown in Figure 1H, the FTIR spectra of native myocardium and dECM exhibited highly comparable spectral profiles, indicating that the decellularization process largely preserved the biochemical composition of the myocardial ECM. The characteristic absorption band observed at approximately 1632 cm^−1^ corresponds to the amide I (C=O stretching) vibration, while the broad band around 3300 cm^−1^ is attributed to the amide A (N–H stretching) vibration, both of which are characteristic of collagen-rich extracellular matrix proteins. The preservation of these characteristic amide bands, together with the overall spectral similarity between native and decellularized tissues, further confirms that the decellularization protocol effectively maintained the structural biochemical integrity of the myocardial ECM while removing cellular components.

### 3.2. Antibacterial Effects of Postbiotics

**Figure 2:**
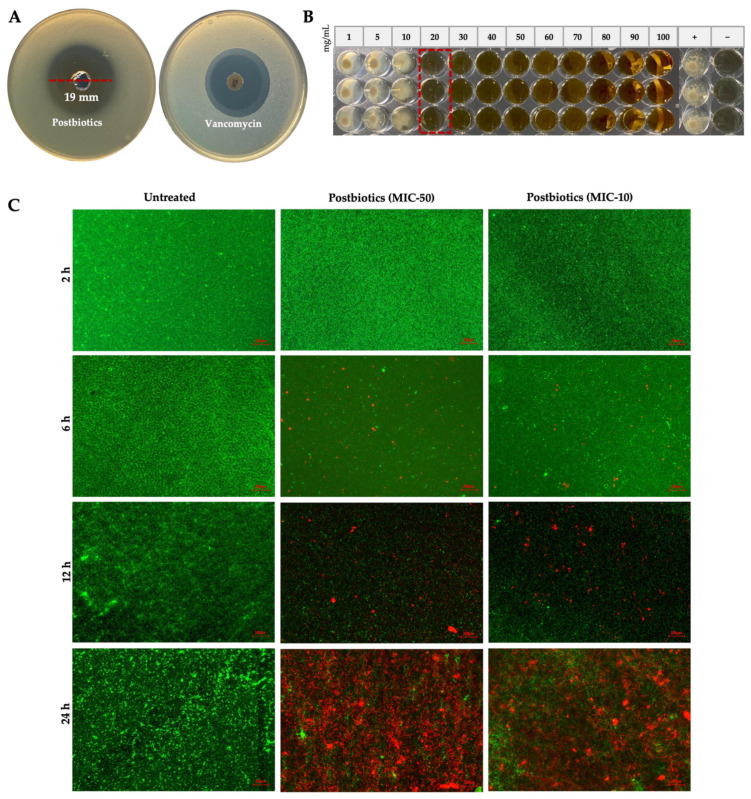
Antibacterial activity of postbiotics against MRSA. (**A**) Agar well diffusion assay showing inhibition zones. (**B**) Broth microdilution assay performed in triplicate demonstrating 20 mg/mL as the MIC value (+, only MRSA; − only media). (**C**) SYTO 9/PI-based Live/Dead fluorescence staining demonstrating time-dependent antibacterial effects of sub-MIC and MIC-10 concentrations. Green fluorescence indicates viable bacterial cells, whereas red fluorescence represents dead bacterial cells (scale bar = 200 μm).

The antibacterial activity of obtained postbiotics against MRSA was initially assessed using the agar well diffusion assay. The obtained results demonstrated that the postbiotic exhibited a prominent antibacterial effect with an inhibition zone diameter of 19 ± 1.4 mm, comparable to the vancomycin control group (19 ± 0.2 mm) (Figure 2A). To investigate the nature of the antimicrobial activity, postbiotic samples were additionally treated with proteinase K or neutralized with 1 N NaOH prior to application. Neutralization markedly reduced the antibacterial activity against MRSA, whereas proteinase K treatment did not substantially affect inhibition zone formation.

Furthermore, broth microdilution analysis revealed that the MIC value of the postbiotic against MRSA was 20 mg/mL (Figure 2B). The antibacterial efficacy of the postbiotic at sub-MIC (10 mg/mL) and MIC-10 (2 mg/mL) concentrations was further evaluated using SYTO 9/PI-based Live/Dead fluorescence staining (Figure 2C).

Fluorescence imaging demonstrated that viable bacterial cells stained predominantly green during the initial 2–6 h incubation period, whereas a marked increase in red fluorescent dead cells was observed after 12–24 h of treatment compared to the untreated control group. Notably, the MIC-10 concentration exhibited a time-dependent antibacterial effect and induced bacterial cell death at a level comparable to that observed in the sub-MIC treatment group after 24 h of incubation.

### 3.3. Cytotoxic Effects of Postbiotics on H9c2 Cells

**Figure 3:**
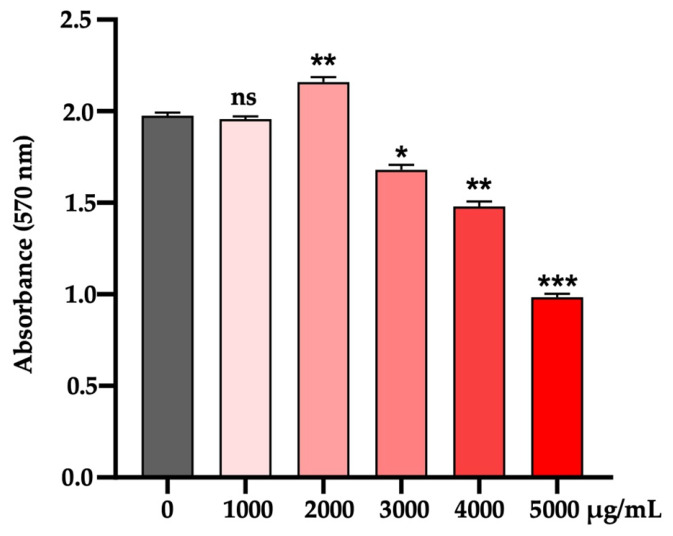
Cytotoxic effects of postbiotics on H9c2 cells assessed by MTT assay (ns: non-significant, *p* > 0.05; * *p* < 0.05, ** *p* < 0.05, ***; *p* < 0.001 versus control).

The cytotoxic effects of postbiotics were evaluated in H9c2 cells using the MTT assay. The results demonstrated that a concentration of 1000 µg/mL did not significantly affect cell viability compared to the untreated group (*p* > 0.05). In contrast, treatment with 2000 µg/mL resulted in a statistically significant increase in cell viability (11 ± 0.6%; *p* < 0.01). At higher concentrations (≥3000 µg/mL), a dose-dependent response was observed, characterized by a progressive and statistically significant reduction in cell viability compared to the untreated group (Figure 3).

### 3.4. Immunomodulatory Effects of Postbiotics on H9c2 Cells

**Figure 4:**
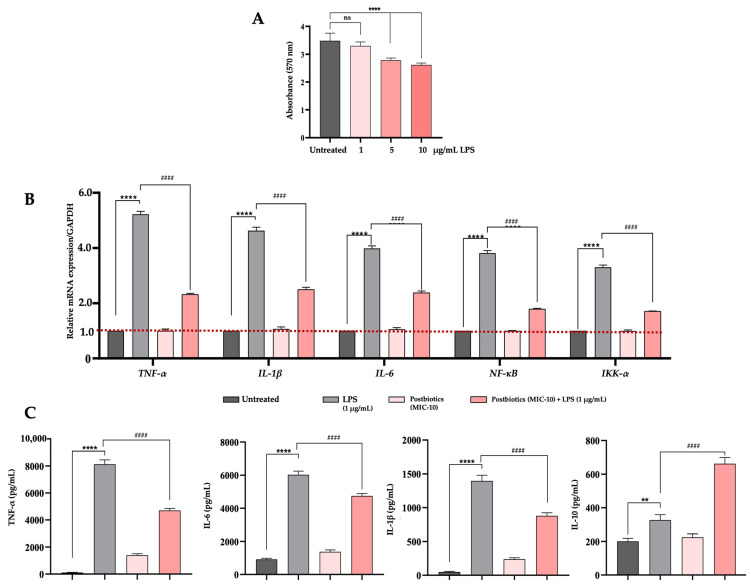
Effects of postbiotics on LPS-induced inflammatory response in H9c2 cells. (**A**) Determination of non-cytotoxic concentration of LPS. (**B**) Relative gene expression of pro-inflammatory (TNF-α, IL-1β, IL-6) and anti-inflammatory (IL-10) cytokines together with (**C**) ELISA quantification of inflammatory mediators following postbiotic pretreatment. (ns: non-significant, *p* > 0.05; ** *p* < 0.01 versus control; **** *p* < 0.0001 versus control; #### *p* < 0.0001 versus model).

The immune response of postbiotics in H9c2 cells was evaluated under LPS-induced inflammatory stress. Prior to stimulation, the highest non-cytotoxic concentration of LPS in H9c2 cells was determined as 1 µg/mL based on MTT assay results and was used in all subsequent experiments (Figure 4A). Following stimulation, qRT-PCR analysis demonstrated that no significant inflammatory response was observed in the negative control group (*p* > 0.05). In contrast, pre-treatment with postbiotics (2000 µg/mL) significantly modulated inflammatory gene expression in LPS-stimulated cells. Specifically, *IL-6* expression was reduced by 1.67-fold (*p* < 0.001), *IL-1β* by 1.85-fold (*p* < 0.0001), *TNF-α* by 2.25-fold (*p* < 0.0001), *NF-κB* by 2.13-fold (*p* < 0.0001) and *IKK-α* by 1.93-fold (*p* < 0.0001) compared with the LPS-only group. Notably, postbiotics alone did not induce any significant changes in inflammatory gene expression under basal conditions (*p* > 0.05) (Figure 4B). 

At the protein level, a marked suppression of pro-inflammatory cytokines was observed, accompanied by a strong induction of anti-inflammatory signaling (Figure 4C). In comparison with the LPS-only group, TNF-α levels were significantly reduced 1.73-fold (*p* < 0.0001). Similarly, IL-6 levels were decreased by 1.27-fold (*p* < 0.0001), while IL-1β levels exhibited a consistent 1.59-fold reduction (*p* < 0.0001). In contrast, IL-10 levels were markedly elevated in the postbiotic pre-treated group, showing a 2.03-fold increase compared with the LPS-only group (*p* < 0.0001), indicating a clear shift toward an anti-inflammatory cytokine profile.

### 3.5. Antioxidant Effects of Postbiotics

**Figure 5:**
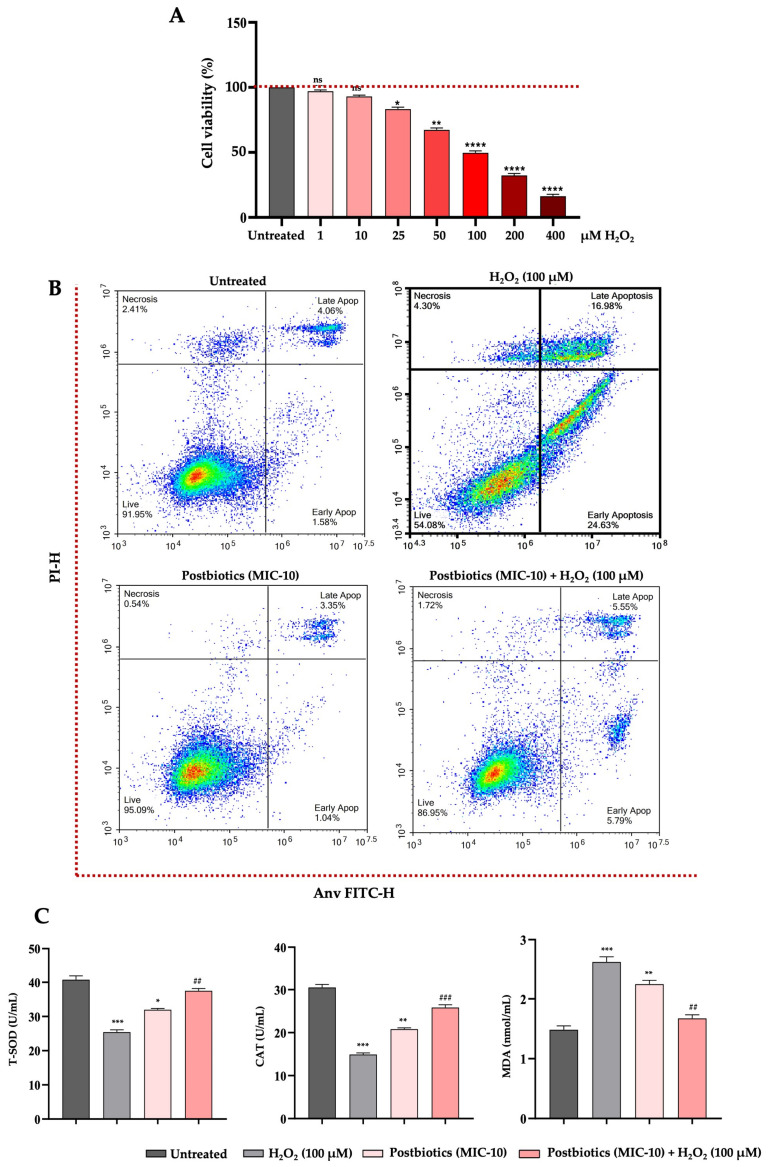
Protective effects of postbiotics against H_2_O_2_-induced oxidative stress in H9c2 cells. (**A**) Determination of the IC_50_ concentration of H_2_O_2_. (**B**) Annexin V–FITC/PI flow cytometry dot plots showing the distribution of viable, early apoptotic, late apoptotic, and necrotic cell populations. (**C**) Biochemical analysis of antioxidant defense markers (ns: non-significant, *p* > 0.05; * *p* < 0.05, ** *p* < 0.01, *** *p* < 0.001, **** *p* < 0.0001 versus untreated group; ## *p* < 0.01, ### *p* < 0.001 versus H_2_O_2_-treated group).

The antioxidant potential of the postbiotics was first evaluated through total phenolic content, total flavonoid content, and DPPH radical scavenging assays. The results demonstrated that the postbiotic contained 5.7 mg/g gallic acid equivalent of total phenolics and 0.84 mg/g quercetin equivalent of total flavonoids. In addition, a strong free radical scavenging activity was observed, with a DPPH inhibition rate of 88.65 ± 2.1%, indicating substantial intrinsic antioxidant capacity. The protective effects of postbiotics against oxidative stress were evaluated using an H_2_O_2_-induced injury model in H9c2 cells. Initial MTT analyses demonstrated that H_2_O_2_ exposure reduced cell viability in a concentration-dependent manner, and the IC_50_ value was determined as 100 µM, corresponding to a 51.07 ± 1.25% reduction in viability (Figure 5A).

Accordingly, this concentration was selected for subsequent oxidative stress experiments. Flow cytometric Annexin V/PI analysis further demonstrated that H_2_O_2_ exposure markedly increased apoptotic cell populations compared with the untreated group. Specifically, viable cell populations decreased from 91.95% in the untreated group to 54.08% following H_2_O_2_ treatment, whereas early and late apoptotic populations were substantially elevated. In contrast, postbiotic pretreatment significantly protected H9c2 cells against oxidative injury, increasing viable cell populations to 86.95% while markedly reducing both early and late apoptosis rates. Moreover, postbiotics alone did not induce detectable cytotoxic or apoptotic effects, with cell viability remaining comparable to the untreated group (Figure 5B). Consistent with these findings, biochemical analyses revealed that H_2_O_2_ exposure significantly impaired endogenous antioxidant defense systems, as evidenced by reduced T-SOD and CAT activities together with elevated MDA levels, indicating enhanced lipid peroxidation. Compared with the untreated group, H_2_O_2_ treatment decreased T-SOD and CAT activities by approximately 1.60-fold and 2.03-fold, respectively, while increasing MDA levels by nearly 1.75-fold. In contrast, postbiotic pretreatment significantly restored antioxidant enzyme activities, resulting in 1.48-fold and 1.73-fold increases in T-SOD and CAT levels, respectively, compared with the H_2_O_2_-treated group. Moreover, MDA accumulation was reduced by approximately 1.53-fold following postbiotic pretreatment (Figure 5C).

### 3.6. Induction of Collagen Synthesis

**Figure 6:**
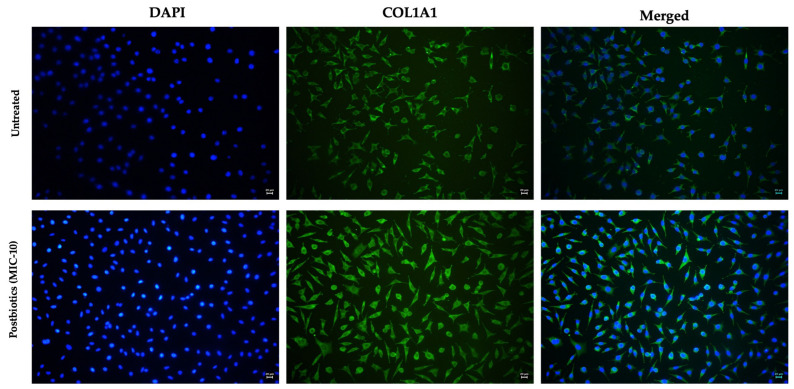
Immunofluorescence images showing COL1A1-positive signals (green) and DAPI-stained nuclei (blue) in untreated and postbiotic-treated groups (scale bar = 20 μm).

The effects of postbiotics on collagen synthesis were evaluated at both the gene expression and cellular levels. Treatment of H9c2 cells with 2000 μg/mL of postbiotics for 24 h resulted in a significant upregulation of *COL1A1* mRNA expression by 1.48-fold (*p* < 0.01) compared to untreated cells. Immunofluorescence analysis further supported these findings at the cellular level. COL1A1-positive signals were markedly increased in postbiotic-treated cells compared with the control group, indicating enhanced collagen type I production (Figure 6).

### 3.7. Architecture and Functional Properties of Electrospun Hybrid Patches

**Figure 7:**
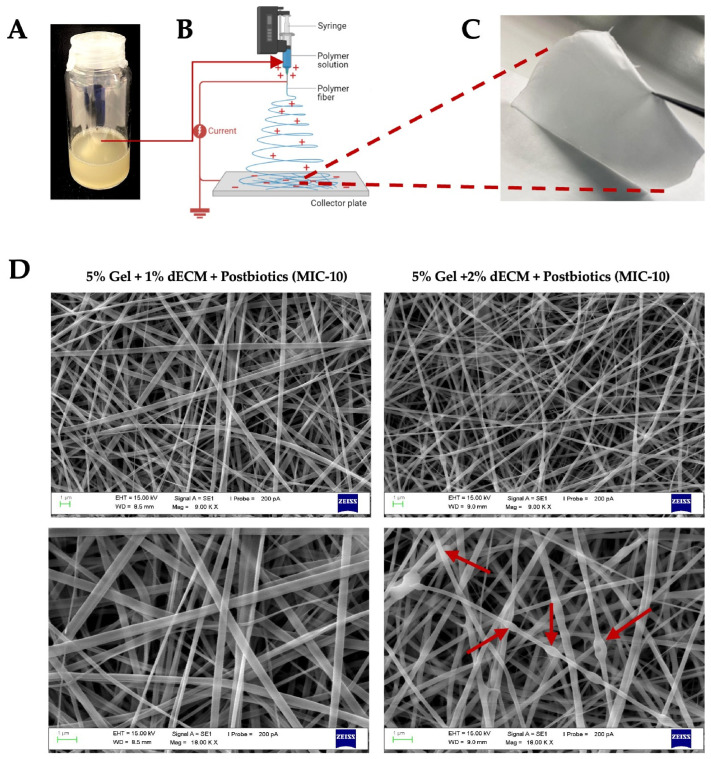
Fabrication and morphological characterization of electrospun hybrid cardiac patches. (**A**) Homogeneous electrospinning precursor solution containing gelatin, myocardial dECM, and postbiotics. (**B**) Schematic illustration of the electrospinning process used for fabrication of hybrid cardiac patches (Figure created using BioRender.com). (**C**) Macroscopic appearance of the fabricated electrospun patch following crosslinking. (**D**) SEM micrographs of electrospun hybrid patches containing 5% gelatin with either 1% or 2% myocardial dECM and postbiotics (red arrows indicate fused fiber regions and bead-like structural irregularities).

Electrospun cardiac patches were successfully fabricated using gelatin, neonatal porcine myocardial dECM, and postbiotics through a stable electrospinning process (Figure 7A–C). SEM analyses demonstrated that both scaffold formulations exhibited highly interconnected and porous nanofibrous architectures with relatively homogeneous fiber distribution, indicating the successful formation of biomimetic ECM-like structures. Moreover, the incorporation of postbiotics did not adversely affect fiber continuity or scaffold integrity. 

Quantitative analysis of the SEM micrographs revealed that the average fiber diameter was 405.5 ± 154.4 nm for the scaffold containing 1% dECM and 338.4 ± 87.0 nm for the scaffold containing 2% dECM. Although the 2% dECM formulation exhibited a smaller average fiber diameter and a narrower fiber diameter distribution, it also showed localized fiber fusion and occasional bead formation. In contrast, the scaffold containing 1% dECM displayed a continuous, uniform, and well-organized nanofibrous morphology. Quantitative pore size analysis further demonstrated mean pore sizes of 654.7 ± 175.3 nm and 668.3 ± 172.8 nm for the 1% and 2% dECM formulations, respectively, indicating comparable pore architectures between the two scaffold compositions. Based on its superior morphological characteristics and the absence of structural defects, the scaffold containing 1% dECM was selected for all subsequent experiments.

**Figure 8:**
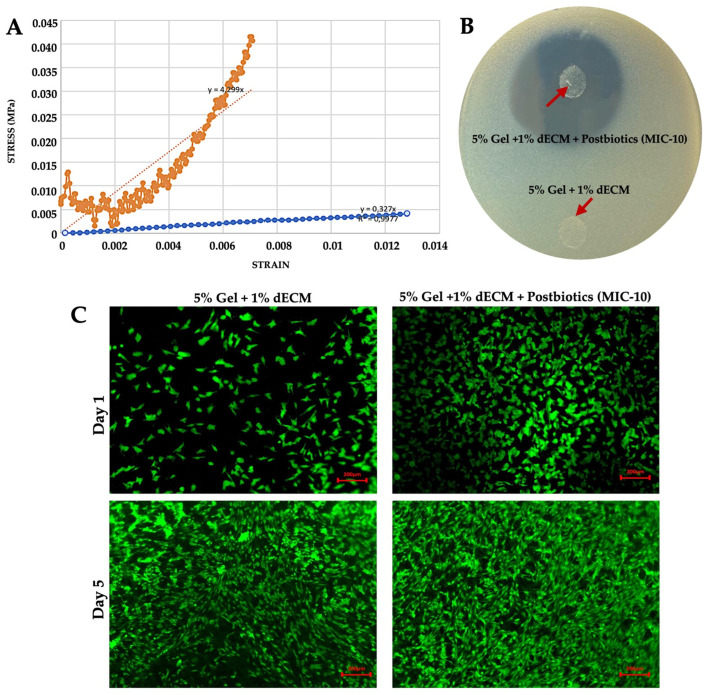
Functional characterization and biocompatibility of electrospun hybrid cardiac patches. (**A**) Representative stress–strain curves of electrospun cardiac patches with (orange) and without (blue) postbiotic and dECM incorporation. (**B**) Antibacterial activity of patches against MRSA evaluated by disk diffusion assay (red arrows indicate patches). (**C**) Live/Dead fluorescence images of H9c2 cells on the electrospun cardiac patches (green, viable cells; red, dead cells; scale bar = 200 μm).

Following the successful fabrication of the electrospun cardiac patches, comprehensive characterization tests were performed to evaluate their mechanical properties, swelling behavior, and degradation profiles. Mechanical characterization demonstrated that incorporation of postbiotics and dECM improved the tensile behavior of the electrospun cardiac patches, as evidenced by increased stress resistance and enhanced elasticity compared with the control group (Figure 8A). 

The maximum tensile strength of the hybrid scaffold was determined to be 0.041 ± 0.002 MPa, whereas the maximum strength value of the control group was found to be 0.004 ± 0.001 MPa. In addition, the Young’s modulus values were calculated as 4.30 ± 0.12 MPa for the hybrid scaffold and 0.33 ± 0.05 MPa for the control group, indicating that the hybrid structure exhibited superior mechanical strength and elastic properties.

Swelling analysis revealed rapid water uptake behavior, with the electrospun cardiac patches reaching equilibrium swelling within 24 h. The maximum swelling ratio was calculated as 310 ± 18%, reflecting the strong hydrophilic nature of gelatin and dECM components. This property supports effective hydration and diffusion of nutrients in a biological environment. Biodegradation studies demonstrated a gradual enzymatic degradation profile in collagenase type I solution. After 7 days, residual mass values decreased to 28.3 ± 2.7%, indicating a controlled degradation rate compatible with tissue remodeling processes.

In addition, antibacterial analyses revealed that postbiotic-loaded patches exhibited a distinct inhibition zone against MRSA, whereas the scaffold lacking postbiotics did not show notable antibacterial activity (Figure 8B). This finding confirmed that the incorporated postbiotics preserved their antimicrobial functionality following electrospinning and crosslinking processes. Functionally, biological assays confirmed that DPPH radical scavenging activity reached 87.4 ± 1.5%.

Biocompatibility analysis further demonstrated that H9c2 cells successfully adhered to and proliferated on the scaffold surfaces over the culture period (Figure 8C). Live/Dead staining showed predominantly viable (green fluorescent) cells with minimal dead cell populations at both time points. Moreover, cell density and spreading were markedly enhanced by day 5, particularly in postbiotic-containing electrospun cardiac patches, suggesting that the hybrid microenvironment effectively supported cellular attachment, viability, and proliferation.

## 4. Discussion

Cardiac patches have emerged as promising therapeutic platforms for myocardial regeneration owing to their capacity to provide structural support, enhance cell retention, and promote functional recovery following cardiac injury [3,4]. However, conventional synthetic patch materials are fundamentally constrained by limited bioactivity, inadequate biomimetic fidelity, suboptimal cell–material interactions, and insufficient integration with native myocardial tissue [42]. To address these limitations, decellularized cardiac ECM-based patches have gained considerable attention in myocardial tissue engineering, primarily due to their capacity to conserve native tissue-specific biochemical composition, hierarchical architecture, and endogenous signaling motifs essential for coordinated cellular organization and myocardial remodeling processes [43]. Within this framework, neonatal porcine myocardial dECM has been increasingly recognized as a particularly advantageous biomaterial for cardiac repair, attributable to its favorable immunocompatibility, conserved cardiac-specific matrix composition, and intrinsic pro-regenerative microenvironment [13,44]. Accordingly, neonatal porcine myocardial-derived dECM was selected in the present study as the primary bioactive constituent for the fabrication of electrospun hybrid cardiac patches. To evaluate whether the obtained myocardial dECM retained its structural and biological integrity after decellularization, a multimodal characterization strategy was employed. Following decellularization, myocardial tissues exhibited a marked macroscopic transition from a native reddish phenotype to a whitish translucent morphology, reflecting efficient depletion of cellular and hemoprotein-associated constituents while preserving the gross ECM framework. This qualitative observation was substantiated by a significant reduction in DNA content and the effective removal of nuclear and cytoplasmic remnants. Importantly, residual DNA levels below 50 ng/µL are widely regarded as a benchmark indicative of adequate decellularization and reduced immunogenic burden, thereby supporting the biological safety of the derived dECM scaffolds [45]. In agreement with prior reports on myocardial dECM systems [43,46], histological and immunofluorescence analyses demonstrated that the decellularization protocol preserved the collagen-dominant ultrastructural network of the myocardial ECM while effectively eliminating cellular constituents. The preservation of collagen integrity is particularly critical, as collagen fibers not only provide mechanical stability but also regulate cellular adhesion and tissue remodeling-associated signaling pathways [47]. Collectively, these findings indicate that the obtained dECM retains the essential structural and bio-functional attributes required for downstream fabrication of myocardial tissue-engineered cardiac patches.

Beyond the successful establishment of an ECM-derived structural platform, there is an increasing demand for cardiac patches engineered with functional bioactivity capable of actively modulating the hostile microenvironment associated with MI [4]. This need arises from the fact that post-infarction cardiac remodeling is not solely a consequence of structural loss but rather reflects a multifactorial pathological cascade characterized by excessive inflammation, oxidative stress, apoptotic cell death, and dysregulated ECM turnover [48]. Accordingly, structural support alone is insufficient to restore functional myocardial integrity. In response to these challenges, a variety of bioactive mediators have been incorporated into cardiac patch systems with the aim of enhancing angiogenesis, immunomodulation, and cardiomyocyte survival [3,7,8,9]. Nevertheless, despite the increasing recognition of the gut–heart axis as an important contributor to cardiovascular pathophysiology [14,15], the integration of microbiota-derived postbiotics into cardiac tissue engineering platforms remains largely unexplored. In this context, the present study addresses this critical gap by incorporating microbiota-derived postbiotics into electrospun cardiac patches, thereby bridging emerging mechanistic insights of the gut–heart axis with the well-documented bio-functional properties of postbiotics, including antioxidant, anti-inflammatory, antibacterial, and cytoprotective activities. Importantly, although a limited number of studies have reported the incorporation of postbiotic-derived components into electrospun scaffolds, their use has predominantly been confined to antimicrobial systems and wound dressing platforms [23,24,25,26,27]. Notably, these approaches have not addressed the specific pathophysiological complexity of myocardial infarction or the requirements of cardiac tissue reconstruction. Therefore, to the best of our knowledge, this work represents the first study to integrate microbiota-derived postbiotics into an electrospun cardiac patch platform specifically engineered for myocardial tissue engineering applications.

Within the scope of the present study, the biological activities of the postbiotics intended for incorporation into the cardiac patches were systematically investigated to optimize scaffold functionality in a therapeutically relevant manner. Among the evaluated properties, the postbiotics demonstrated pronounced antibacterial activity against MRSA, one of the most clinically challenging pathogens associated with hospital-acquired infections. Importantly, the observed antibacterial efficacy was largely attributed to the production of organic acids, as neutralization of the postbiotic preparations markedly attenuated their inhibitory activity, indicating a pH-dependent antimicrobial mechanism. Furthermore, fluorescence-based Live/Dead analyses revealed a progressive increase in bacterial cell death following postbiotic exposure, suggesting sustained bactericidal activity rather than a transient bacteriostatic response. Similar antibacterial profiles have likewise been reported in postbiotic formulations derived from various probiotic strains, further supporting the broad-spectrum antimicrobial potential of postbiotic metabolites [49,50]. From a translational perspective, these findings are particularly relevant in the context of cardiovascular biomaterials, where MRSA-related infections remain a major cause of post-surgical complications, implant failure, prolonged hospitalization, and increased mortality. Accordingly, the incorporation of antibacterial postbiotics into cardiac patch systems may provide dual therapeutic functionality by simultaneously supporting myocardial regeneration while establishing a biologically protective microenvironment against infection-associated complications following implantation.

In addition to their antibacterial activity, the postbiotics demonstrated pronounced anti-inflammatory effects under both LPS-induced inflammatory stress conditions in H9c2 cardiomyoblast cells, further underscoring their multifunctional therapeutic potential. Mechanistically, LPS stimulation activates TLR4-dependent signaling cascades, triggering phosphorylation of the IKK complex, subsequent NF-κB activation [51], and excessive secretion of pro-inflammatory cytokines that collectively drive myocardial injury, adverse ventricular remodeling, and cardiomyocyte dysfunction following myocardial infarction [52]. Consistent with this mechanism, postbiotic pretreatment markedly suppressed the expression of key inflammatory mediators, including IL-6, IL-1β, TNF-α, IKK-α, and NF-κB, while simultaneously enhancing IL-10 production at the protein level, indicating a potent immunomodulatory effect directed toward restoration of inflammatory homeostasis. Importantly, postbiotics alone did not induce inflammatory activation under basal conditions, suggesting that their regulatory activity is associated with selective attenuation of pathological inflammatory signaling rather than nonspecific immune stimulation. These findings are in agreement with previous reports demonstrating that microbiota-derived postbiotics can suppress NF-κB-dependent inflammatory pathways [53] and reduce pro-inflammatory cytokine overproduction [54] across various inflammatory disease models [55]. Taken together, the obtained results suggest that the incorporation of postbiotics into cardiac patch systems may provide an additional immunoregulatory functionality capable of mitigating inflammation-associated myocardial damage while simultaneously establishing a more favorable microenvironment for post-infarction cardiac repair.

Building upon their previously demonstrated immunomodulatory effects under LPS-induced inflammatory stress, postbiotics further exhibited pronounced cytoprotective and antioxidant activities against H_2_O_2_-mediated injury in H9c2 cardiomyoblast cells. This dual activity is particularly relevant in the context of myocardial injury, where oxidative stress and NF-κB-driven inflammatory signaling are tightly interconnected and mutually reinforcing pathological processes [56]. Accordingly, the simultaneous attenuation of NF-κB-associated inflammatory mediators alongside protection against oxidative damage suggests that postbiotics may act through coordinated modulation of redox-sensitive signaling networks rather than isolated pathway regulation. However, confirmation of this mechanism requires protein-level analyses, such as Western blotting or immunofluorescence. In line with this mechanistic interpretation, previous studies have reported that microbiota-derived postbiotics can suppress NF-κB activation while concurrently enhancing cellular antioxidant capacity [54,57]. Consistent with these findings, postbiotic pretreatment restored endogenous antioxidant defenses, as evidenced by increased T-SOD and CAT activities together with reduced MDA accumulation, indicating attenuation of lipid peroxidation and preservation of intracellular redox homeostasis. Similar enhancements in antioxidant enzyme systems, particularly SOD- and CAT-associated pathways, have been widely reported in studies investigating microbiota-derived postbiotics, where reinforcement of enzymatic antioxidant capacity was identified as a key mechanism underlying cytoprotection under oxidative stress conditions [58,59]. These enzymatic effects are likely associated with the intrinsic biochemical composition of the postbiotics, particularly their phenolic- and flavonoid-rich content, which has previously been linked to potent radical scavenging activity and modulation of redox-sensitive signaling cascades in various microbiota-derived postbiotic formulations [60,61]. Collectively, these findings suggest that the incorporation of postbiotics into cardiac patch systems may provide simultaneous antioxidative functions capable of mitigating oxidative myocardial damage and promoting a more favorable microenvironment for cardiac repair following implantation.

Given that type I collagen represents a principal structural component of the myocardial ECM and plays a central role in maintaining tissue integrity as well as orchestrating post-injury remodeling, its tightly regulated expression is critical for effective cardiac repair. In this context, insufficient collagen deposition may compromise mechanical stability, whereas excessive accumulation is closely associated with fibrotic remodeling and impaired cardiac function [62]. Consistent with this biological framework, the present study demonstrated that postbiotic treatment significantly upregulated *COL1A1* expression and increased COL1A1-positive immunofluorescence intensity in H9c2 cardiomyoblast cells, indicating an active modulation of ECM-associated responses. Notably, the observed increase remained moderate, suggesting a controlled and potentially adaptive remodeling response rather than a fibrotic shift. Mechanistically, this effect may be linked to the capacity of postbiotics to attenuate oxidative stress and suppress inflammatory signaling pathways, particularly NF-κB-dependent cascades, which are well recognized as key regulators of fibroblast activation and collagen turnover [63]. Through the reduction of these stress-associated signals, a more permissive microenvironment for balanced ECM remodeling and cellular survival may be established. In agreement with these findings, previous studies have also reported that microbiota-derived postbiotics can modulate collagen synthesis and ECM dynamics [21], collectively suggesting that postbiotics may contribute to the formation of a pro-regenerative ECM milieu with enhanced structural continuity with the host myocardium, thereby ultimately supporting more effective myocardial repair following implantation.

Following the comprehensive evaluation of the biological activities of postbiotics, these bioactive components were subsequently incorporated into a composite scaffold system composed of neonatal porcine myocardial tissue-derived dECM and gelatin for cardiac patch fabrication. In this context, electrospinning was selected as the fabrication strategy due to its extensive use in tissue engineering, providing a robust and versatile platform for the development of hybrid biomaterial constructs, where structural mimicry and microenvironmental fidelity are critical for supporting cell adhesion, survival, and tissue integration [64]. Consistent with these advantages, SEM analysis confirmed the successful formation of a homogeneous and highly interconnected porous fibrous network without bead defects. After fabrication, comprehensive physicochemical and mechanical characterization was performed. From a mechanical standpoint, the Young’s modulus of the fabricated cardiac patch was determined to be ~4.01 MPa, placing it within the upper range of previously reported electrospun and ECM-based cardiac patches. In the literature, electrospun patches for cardiac tissue engineering exhibit a wide mechanical spectrum, thus covering both sub-physiological and supra-physiological regimes of myocardial stiffness [65]. For instance, Kai et al. reported that a poly(ε-caprolactone)/gelatin patch with a Young’s modulus of 1.45 MPa enhanced angiogenesis and improved cardiac function in a myocardial infarction model [66]. Similarly, Guex et al. demonstrated that MSC-seeded functionalized poly(ε-caprolactone) scaffolds with moduli of 16–18 MPa stabilized cardiac function and reduced ventricular dilation in infarcted hearts [67]. Given that the Young’s modulus of healthy human diastolic myocardium is approximately 8–15 kPa [68], it has been shown that different scaffold systems exhibit variable Young’s modulus depending on their composition, but significant findings have been obtained in terms of target efficacy. Therefore, when considered within the broader context of the literature, the present findings further emphasize that cardiac scaffold design should not be guided solely by the pursuit of high mechanical strength.

Collectively, beyond the mechanical and structural performance of the developed system, the biological activity of the fabricated cardiac patch further underscores its functional relevance for myocardial tissue engineering. In particular, the preservation of postbiotic-associated bioactivity following incorporation into the dECM/gelatin electrospun matrix is critical, as it enables the scaffold to extend its role beyond passive structural support toward an active bio-functional interface. In agreement with recent advances in bioactive cardiac scaffolds, previous studies have similarly emphasized that the incorporation of bioactive agents into electrospun ECM-based constructs significantly enhances cellular responses and improves regenerative outcomes compared with purely structural biomaterials [69]. Accordingly, the ability of the present system to retain postbiotic functionality within a stable fibrous architecture aligns with emerging literature highlighting the importance of preserving bioactive molecule stability during scaffold fabrication to ensure sustained therapeutic efficacy [23,24,25,26,70].

In conclusion, this study successfully developed a multifunctional electrospun cardiac patch by integrating decellularized neonatal porcine myocardial dECM with microbiota-derived postbiotics, thereby combining tissue-specific structural biomimicry with biologically active therapeutic functionality. The developed biohybrid platform exhibited favorable physicochemical characteristics together with antibacterial, antioxidant, anti-inflammatory, and cytoprotective properties, highlighting its potential to modulate multiple pathological processes associated with myocardial infarction. These findings demonstrate that the incorporation of microbiota-derived postbiotics into dECM-based electrospun scaffolds represents a promising strategy for enhancing the therapeutic performance of cardiac patches and advancing next-generation myocardial tissue engineering. Nevertheless, the present study is limited by its predominantly in vitro design and the absence of in vivo functional validation. Therefore, future studies should focus on elucidating the molecular mechanisms underlying the biological effects of postbiotics and the comprehensive metabolomic characterization of their active components and evaluating the long-term therapeutic efficacy, biocompatibility, and regenerative potential of the developed cardiac patch in relevant myocardial infarction animal models. Successful completion of these studies will be essential for facilitating the clinical translation of this multifunctional biohybrid platform for regenerative cardiovascular medicine.

## Figures and Tables

**Table 1:** Primers ^1^ used in this study.

Genes	Forward Primer (5′-3′)	Reverse Primer (5′-3′)
*IL-1β*	ACAGCAATGGTCGGGACATA	AGACTGCCCATTCTCGACAA
*IL-6*	CTGCTCTGGTCTTCTGGAGT	AGAGCATTGGAAGTTGGGGT
*TNF-α*	ACCAGGAGAAAGTCAGCCTC	GCTGGGTAGAGAACGGATGA
*NF-κB*	GCACACCTTGATCCAAAGCA	TCAAACCAAACAGCCTCACG
*IKK-α*	GGGAACGTCAGTCTGTACCA	TCAGGAACATCACAGGCCTT
*GAPDH*	GAGACAGCCGCATCTTCTTG	TGACTGTGCCGTTGAACTTG
*COL1A1*	GACGCCATCAAGGTCTACTG	ACGGGAATCCATCGGTCA

^1^ Specific primers were designed using Primer3 software (v0.4.0) and validated via NCBI Primer BLAST tool.

## Data Availability

The original contributions presented in this study are included in the article. Further inquiries can be directed to the corresponding author.

## Abbreviations

HCl	Hydrochloric Acid
NaCl	Sodium Chloride
NaOH	Sodium Hydroxide
PBS	Phosphate-buffered saline
FITC	Fluorescein Isothiocyanate
DAPI	4′,6-diamidino-2-phenylindole
LPS	Lipopolysaccharide
SYTO	Green fluorescent nucleic acid stain
COL1A1	Collagen Type I Alpha 1 Chain
IL-1β	Interleukin-1 beta
IL-6	Interleukin-6
IL-10	Interleukin-10
TNF-α	Tumor Necrosis Factor-alpha
NF-κB	Nuclear Factor kappa-light-chain-enhancer of activated B cells
IKK-α	IκB Kinase alpha
TLR	Toll-Like Receptor
GAPDH	Glyceraldehyde-3-Phosphate Dehydrogenase
MSC	Mesenchymal Stem Cell(s)
ATCC	American Type Culture Collection
NCBI	National Center for Biotechnology Information
MTT	3-(4,5-dimethylthiazol-2-yl)-2,5-diphenyltetrazolium bromide assay
ELISA	Enzyme-Linked Immunosorbent Assay
IC50	Half maximal inhibitory concentration (50% inhibitory concentration)
